# Cellulase production using natural medium and its application on enzymatic hydrolysis of thermo chemically pretreated biomass

**DOI:** 10.1007/s13205-016-0465-z

**Published:** 2016-06-21

**Authors:** Shivani Sharma, Vinay Sharma, Arindam Kuila

**Affiliations:** Bioscience and Biotechnology Department, Banasthali University, Banasthali, 304022 Rajasthan India

**Keywords:** Lignocellulosic bioethanol, Cellulase, Thermochemical pretreatment, FTIR, SEM

## Abstract

Lignocellulosic bioethanol is an important renewable fuel for transportation purpose. Commercial production of lignocellulosic bioethanol mainly depends on cost of cellulase production, efficient pretreatment and enzymatic hydrolysis process. In the present study cellulase production from *Aspergillus niger* under submerged fermentation (SmF) was optimized using coconut water as natural medium. Maximum cellulase production (0.53 IU/mL) was achieved within 3 days of incubation using 8 % (w/v) waste paper and 0.07 % (w/v) glucose. The produced cellulase was applied for enzymatic hydrolysis of thermo chemically (dilute acid and alkaline) pretreated biomass (equal mixture of wheat straw and cotton stalk). Optimization of dilute acid and dilute alkaline pretreatment showed dilute alkaline pretreatment was more effective for higher reducing sugar production. Maximum reducing sugar yield of 398.0 mg/g dry biomass was obtained from dilute alkaline pretreated biomass (using 0.5 M sodium hydroxide, 8 % substrate concentration, 120 °C temperature and 20 min of incubation time). The presence of difference sugars (glucose, xylose, mannose, maltose) in the saccharified sample was confirmed by thin layer chromatographic analysis. The effectiveness of dilute alkaline pretreatment was further confirmed by biochemical composition (cellulose, hemicelluloses and lignin) and structural (furrier transformed infrared spectroscopic and scanning electron microscopic) analysis. The above result can be useful for commercial production of lignocellulosic bioethanol.

## Introduction

Biofuel production from lignocellulosic biomass has several attractive features such as high availability, no competition with food chain and abundant in supply. Lignocellulosic biomass mainly composed of cellulose, hemicelluloses and lignin. For biofuel production, there needs hydrolysis of carbohydrates (cellulose and hemicelluloses) portion of lignocellulosic biomass (Khare et al. 2015). Prior to hydrolysis most lignocellulosic substrates need to undergo some sort of pretreatment to enhance the accessibility of the substrate for efficient hydrolysis and biofuel production. Thermo chemical pretreatment is one such process. It has several advantages such as efficient lignin removal within shorter incubation time and high sugar yield (Chen et al. 2013; Singh and Trivedi 2013). Akanksha et al. (2014) reported optimization of dilute acid pretreatment of sorghum biomass. They found maximum reducing sugar yield (0.408 g reducing sugar/g of biomass) when biomass was pretreated using 0.37 % sulphuric acid at 150 °C for 15 min. McIntosh and Vancov (2011), reported enzymatic hydrolysis of dilute alkaline pretreated wheat straw. Pretreating biomass using 2 % sodium hydroxide for 30 min at 121 °C, increased the reducing sugar yield up to 6.3 fold compared to control biomass. After pretreatment, enzymatic hydrolysis is the second step for lignocellulosic biofuel production. Cellulases are used for enzymatic hydrolysis of plant carbohydrate polymers. It is a hydrolytic enzyme that degrades cellulose to glucose. Several authors reported on enzymatic hydrolysis of different types of lignocellulosic biomass (Nitsos et al. 2013; Bals et al. 2014; Maitan-Alfenas et al. 2015). But major drawback on large scale trial of enzymatic hydrolysis of lignocellulosic biomass is the cost of cellulase enzyme. Till now there is no viable technology which can produce cellulase in cost effective manner. For cheaper cellulase production, high cost of medium constituent is major limiting factor. In such case, coconut water can be used as cheaper alternative for higher cellulase production. Major constituents of coconut water are total sugar 32 g/L, glucose 13.5 g/L, protein 5.5 g/L, calcium 7 mmol/L, magnesium 3.4 mmol/L, pH 5.6 (Vigliar et al. 2006; Prades et al. 2012).

Previously several authors worked on cellulase production under solid state fermentation (SSF) in cost effective manner (Gupta et al. 2015; Kuila et al. 2015; Mangalanayaki and Madhavan 2015). Although SSF has several advantages for higher cellulase production, but it has different drawbacks for large scale enzyme production such as require large space for enzyme production, less amount of enzyme are extracted after fermentation, purification of enzyme is difficult etc. In such case, submerged fermentation (SmF) are used for production of several industrially important enzymes (cellulase, xylanase, laccase etc.) due to its several advantages such as greater control of environmental factors (temperature, pH), require less number of space, higher amount of enzyme can be extracted after fermentation, purification of the enzyme is easier.

In the present investigation, equal mixture of wheat straw and cotton stalk (abundantly available in India) were used for optimization of thermo chemical pretreatment (dilute acid and alkaline). After that, enzymatic hydrolysis was carried out using pretreated biomass. Enzyme production was optimized under submerged fermentation (SmF) using natural medium (coconut water) and waste paper. According to our knowledge, this is the first report on cellulase production under SmF using coconut water (highly available in India) as growth medium.

## Materials and methods

### Biomass

Wheat straw and cotton stalk collected from nearby locality of Banasthali University, Rajasthan. Both the substrates were dried overnight at 70 °C. Dried substrates were milled to particle size less than 0.2 mm. After that both the milled substrates were mixed in equal proportion and further used for thermo chemical pretreatment.

### Cellulase production

Cellulase production was carried out under submerged fermentation in 250 ml Erlenmeyer flask which contained 100 mL of sterile medium. The composition of the medium was: coconut water and varying concentration of waste news paper. A small spore suspension (1 × 10^7^ spores/mL) of *Aspergillus niger* MS82 agar slant was added to the 100 mL sterile medium. Cellulase assay (FPase) was carried out by following standard assay protocol (Nathan et al. 2014). The cellulase production experiment was focused on FPase activity, produced by *Aspergillus niger* and further optimized using central composite design (CCD) based response surface methodology (RSM). The parameters and their ranges were: glucose concentration (0.025–0.075 %, w/v), waste news paper concentration (2–8 %, w/v) and incubation time (3–5 days). Total 20 runs were carried out for optimization study. Each experiment was carried out in triplicates. After optimization of cellulase production, it was further used for enzymatic hydrolysis of pretreated substrate.

### Thermo chemical pretreatment of biomass

The mixture of biomass was thermo chemically pretreated using dilute sulphuric acid and sodium hydroxide. For optimization study the parameters varied were: biomass concentration (1–10 %, w/v), sulphuric acid/sodium hydroxide concentration (0.1–1 M) and incubation time (5–40 min). After each type of pretreatment biomass was washed with distilled water and then dried overnight at 70 °C. After that dried biomass was subsequently used for enzymatic hydrolysis experiments.

### Biochemical composition analysis of biomass

Biochemical composition (extractives, cellulose, hemicelluloses and lignin) of raw and optimum pretreated (sodium hydroxide pretreated) biomass were determined by following the procedure of Yang et al. (2006). In this procedure, biomass was extracted with acetone. The amount of extractives was measured as weight difference of the biomass before and after extraction. To determine hemicelluloses content, the extractive free biomass was treated with 0.5 M sodium hydroxide. The weight difference before and after sodium hydroxide treatment was the hemicelluloses content. To determine lignin content, extractive free biomass was treated with sulphuric acid (98 %). The weight difference before and after sulphuric acid treatment was the lignin content. The weight difference of the initial biomass and total lignin, hemicelluloses and extractive content was calculated as cellulose content of the biomass (assuming that biomass contains only cellulose, hemicelluloses, lignin and extractives).

### Enzymatic hydrolysis of pretreated biomass

Enzymatic hydrolysis was carried out under following conditions: pretreated substrate loading: 2.5 %, cellulase enzyme loading: 20 FPU/g dry substrate, temperature: 50 °C and incubation time: 24 h. After enzymatic hydrolysis, samples were withdrawn and centrifuges at 5000 rpm for 10 min. After that supernatant were collected separately and measured for reducing sugar by following miller method (Miller 1959).

### Fourier transformed infrared spectroscopy (FTIR) study

FTIR study was carried out in control and pretreated (sodium hydroxide pretreated) biomass using KBr pellet technique. Sample spectra were taken in the range of 600 and 4000 cm^−1^ with the spectral resolution of 0.5 cm^−1^.

### Field emission scanning electron microscopy (FESEM) study

FESEM (Mira 3, Tescan, field emission scanning electron microscope) was carried out in both the control and pretreated (sodium hydroxide pretreated) biomass. Before FESEM analysis samples were dried and coated with gold.

### Thin layer chromatography (TLC) analysis

TLC analysis of saccharified sample of pretreated biomass (sodium hydroxide pretreated) was carried out using TLC plate. The mobile phage used was ethyl acetate, isopropanol, water and pyridine (26:14:7:2). After complete run plat was dried and sugar spots were detected with aniline diphenylamine reagent. The sugar spots were detected against various standard sugars (glucose, xylose, mannose, maltose, ribose and arabinose). For TLC analysis, samples were prepared in absolute ethanol in a ratio of 3:1 and then centrifuged for the separation of any residual protein.

## Result and discussion

### Optimization of cellulase production using CCD based RSM

Cellulase production under submerged fermentation was optimized using CCD based RSM. Table 1 showed the experimental design and response for cellulase production.

**Table 1:** Experimental design and responses for cellulase production by *Aspergillus niger*

Run order	Glucose concentration (%)	Substrate concentration (%)	Incubation time (days)	Cellulase activity (IU/mL)	
				Experimental	Predicted
1	0.025	2	3	0.28	0.26
2	0.075	2	3	0.30	0.31
3	0.025	8	3	0.34	0.34
4	0.075	8	3	0.51	0.49
5	0.025	2	5	0.41	0.42
6	0.075	2	5	0.16	0.15
7	0.025	8	5	0.37	0.36
8	0.075	8	5	0.18	0.19
9	0.025	5	4	0.38	0.39
10	0.075	5	4	0.33	0.33
11	0.05	2	4	0.37	0.37
12	0.05	8	4	0.42	0.43
13	0.05	5	3	0.38	0.41
14	0.05	5	5	0.35	0.34
15	0.05	5	4	0.40	0.41
16	0.05	5	4	0.41	0.41
17	0.05	5	4	0.42	0.41
18	0.05	5	4	0.42	0.41
19	0.05	5	4	0.43	0.41
20	0.05	5	4	0.40	0.41

Interactive effect of the independent variables (glucose concentration, waste news paper concentration and incubation time) was investigated to obtain optimum conditions of cellulase production. ANOVA analysis (Table 2) carried out that gave following second order polynomial model by response surface regression method (Mukhopadhyay et al. 2011):

**Table 2:** ANOVA of RSM model for for cellulase production by *Aspergillus niger*

Source	DF^a^	Seq SS^b^	Adj SS^b^	Adj MS^c^	*F*	*P*
Regression	9	0.12784	0.12784	0.01421	44.71	<0.001
Linear	3	0.02956	0.05830	0.01943	61.17	<0.001
Square	3	0.03265	0.03265	0.01088	34.25	<0.001
Interaction	3	0.06564	0.06564	0.02188	68.87	<0.001
Residual error	10	0.00318	0.00318	0.00032		
Lack-of-fit	5	0.00244	0.00244	0.00049	03.33	0.106
Pure error	5	0.00073	0.00073	0.00015		
Total	20	0.13102				
*R* ^2^	97.58 %	95.39 %				

^a^Degree of freedom

^b^Sum of squares

^c^Mean squares

1 $$\begin{aligned} {\text{Cellulase activity }}\left( {{\text{IU}}/{\text{mL}}} \right) & = - 1.0 3 4 9 + 1 7. 2 1 \times {\text{glucose concentration}} + 0.0 5 \times {\text{substrate concentration}} \\ & \quad + 0. 4 8 \times {\text{incubation time}} - 7 5. 6 4 \times {\text{glucose concentration}} \times {\text{glucose concentration}} \\ & \quad - 0.0 4 \times {\text{incubation time}} \times {\text{incubation time}} + 0. 3 5 \times {\text{glucoseconcentration}} \\ & \quad \times {\text{substrate concentration}} - 3. 1 5 \times {\text{glucose concentration}} \times {\text{incubation time}} \\ & \quad - 0.0 1 \times {\text{substrate concentration}} \times {\text{incubation time}} \\ \end{aligned}$$

where, cellulase activity (IU/mL) is response, glucose concentration, substrate concentration and incubation time are uncoded independent variables.

From ANOVA Table it was found that the *F* value was 44.71 and *P* value was <0.001 at 9 degree of freedom. The obtained *F* value was lesser than table *F* value and consequent *P* value was very less (less than 0.05), which showed that the RSM model adequately describe the relationship between the response (cellulase activity) and the independent variables. Further, the observed and adjusted regression coefficient (*R* ^2^) values were 97.58 and 95.39 %, respectively. This demonstrated that the present model was capable of describing maximum variation in the data.

**Fig. 1:**
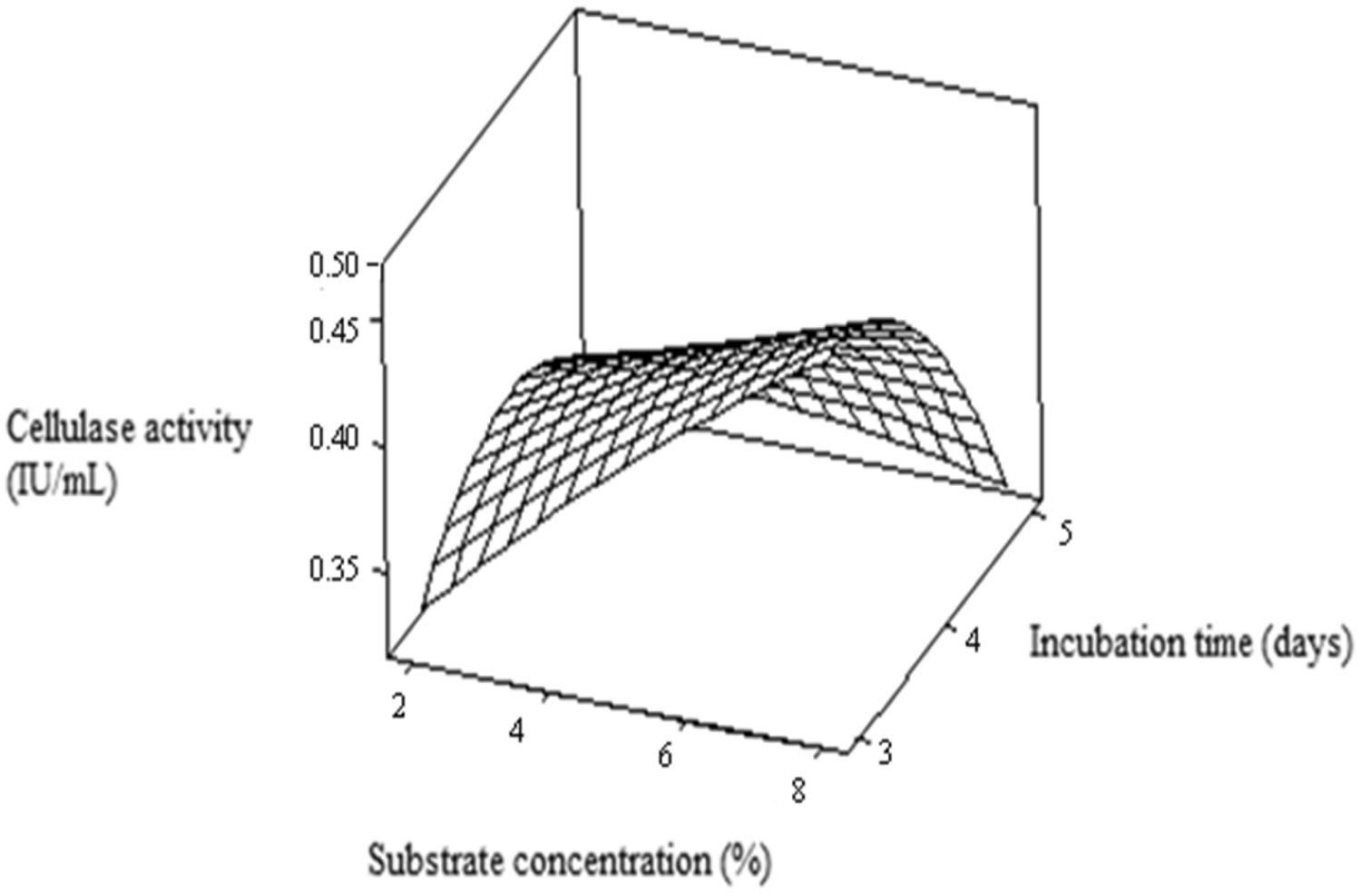
RSM plot showing the effect of substrate concentration (%) and incubation time (days) on cellulase production (IU/mL)

**Fig. 2:**
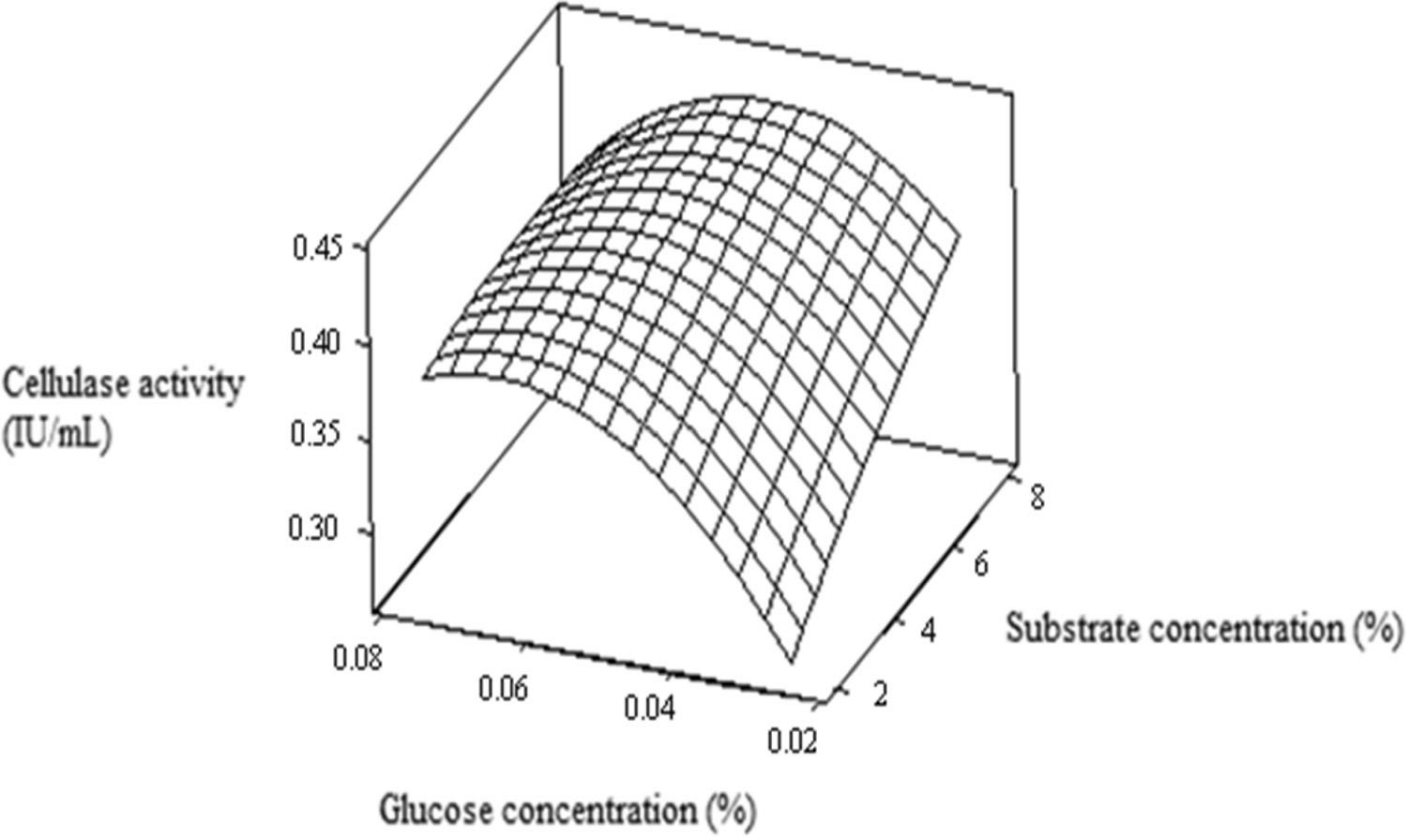
RSM plot showing glucose concentration (%) and substrate concentration (%) on cellulase production (IU/mL)

**Fig. 3:**
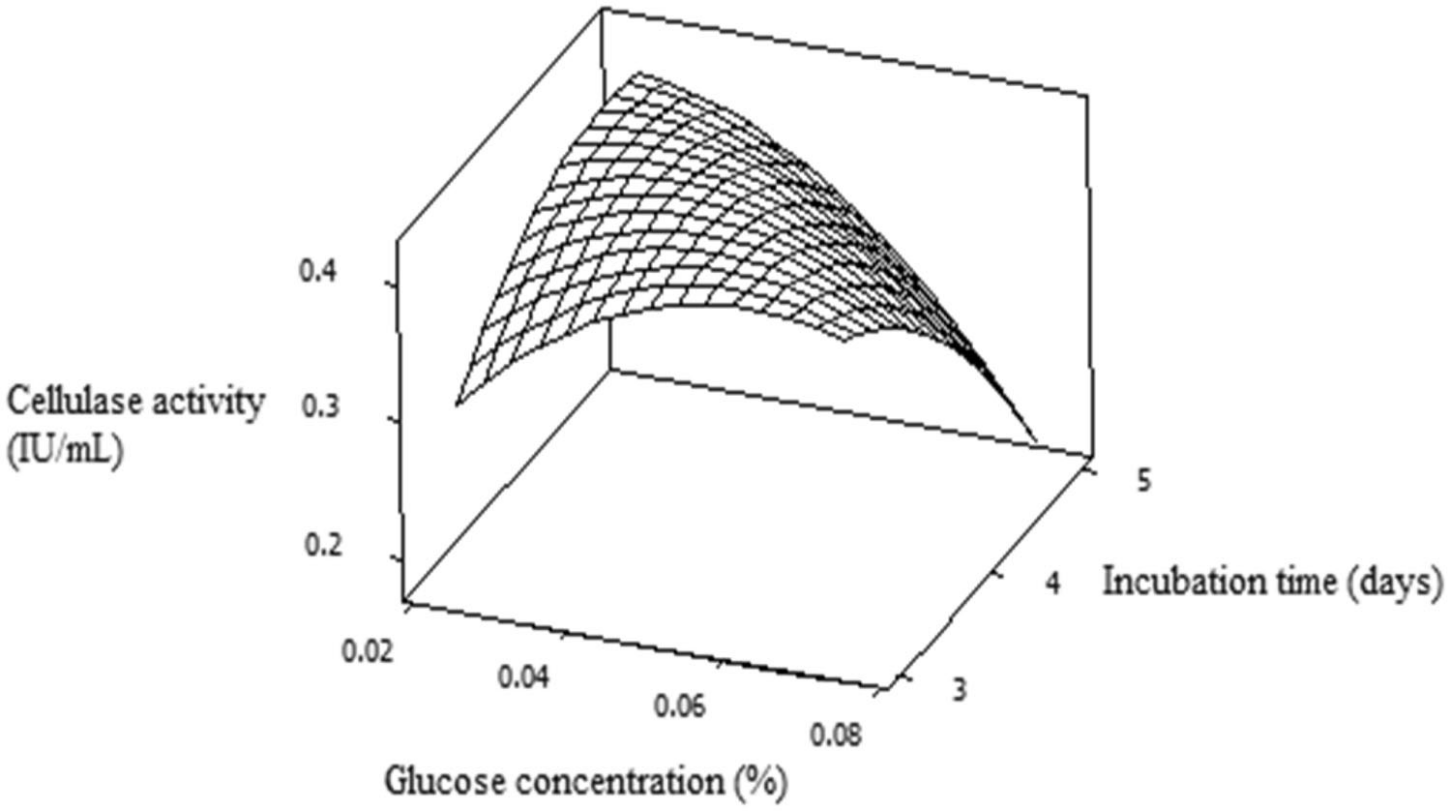
RSM plot showing glucose concentration (%) and incubation time (days) on cellulase production (IU/mL)

The interactive effect of independent variables was observed using 3D response surface plot analysis. Each figure represents the effect of two different independent variables on cellulase production while other parameters kept constant at its optimum point. Figure 1 showed the effect of substrate concentration and incubation time on cellulase production from *Aspergillus niger*. It demonstrated by increasing substrate concentration along with incubation time cellulase activity was increased and maximum cellulase activity (0.53 IU/mL) was obtained using 8 % substrate concentration and after 3 days of incubation time. After 3 days of incubation time, further increase in incubation time cellulase production was decreased significantly. Damisa et al. (2012) reported cellulase production under submerged fermentation using waste paper as substrate. Authors reported maximum cellulase activity (0.18 IU/mL) after 96 h of incubation. The difference in cellulase activity was might be due to different strain and fermentation medium used for cellulase production. Manglanayaki and Madhavan (2015) reported maximum cellulase production (0.76 IU/mL) using 3 % substrate concentration after 9 days of incubation. Figure 2 demonstrated the effect of glucose and substrate concentration on cellulase production. It showed maximum cellulase activity was obtained using 0.07 % (w/v) glucose concentration. Interactive effect of glucose concentration and incubation time on cellulase production has been demonstrated in Fig. 3. It showed by increasing glucose concentration along with incubation time cellulase activity was increased but after certain value further increase or decrease its concentration cellulase production was decreased. From 3D response surface plot analysis, the optimum predicted conditions for cellulase production was: glucose concentration 0.07 % (w/v), substrate concentration 8 % (w/v) and incubation time 3 days. Under above conditions maximum experimental cellulase activity was found to be 0.53 IU/mL, which was very close to predicted response (0.54 IU/mL). Kumar et al. (2011) reported maximum *Aspergillus* cellulase production (0.36 IU/mL) under following conditions: substrate concentration 6.5 %, pH 4.6 and incubation time 126 h. Saini et al. (2015) reported cellulase production from *Penicillium oxalicum* under submerged fermentation. Authors reported maximum cellulase production of 1.2 IU/mL from after 8 days of incubation using complex growth medium. In the present investigation, maximum cellulase production of 0.53 IU/mL was obtained within 3 days incubation time using coconut water as growth medium. For cost effective cellulase production medium constituents and incubation time are the major limiting factors (Gautam et al. 2011). The present investigation can be further studied by gradual scaling up of cellulase production for additional enhancement of enzyme production. After optimization of cellulase production, the cellulase was used for enzymatic hydrolysis of pretreated biomass.

### Biochemical composition of lignocellulosic biomass

Table 3 showed biochemical composition of raw and pretreated (sodium hydroxide pretreated) biomass. It showed that after pretreatment lignin and hemicelluloses content decreased considerably but there was no significant effect on cellulose content. This observation was might be due to selective degradation of lignin and hemicelluloses after alkaline pretreatment. Similar type of observation was reported previously in case of sodium hydroxide treated corn stover (Chen et al. 2013). For enhanced enzymatic hydrolysis, there needs low lignin content and high cellulose content. The present result showed effectiveness of dilute alkaline pretreatment for efficient hydrolysis of lignocellulosic biomass.

**Table 3:** Biochemical composition of lignocellulosic biomass

Biomass type	Cellulose (%)	Hemicellulose	Lignin (%)
Lignocellulosic biomass (raw)	38.5	22.0	18.95
Lignocellulosic biomass (dilute sodium hydroxide pretreated)	40.02	15.06	10.25

### Pretreatment of lignocellulosic biomass

**Fig. 4:**
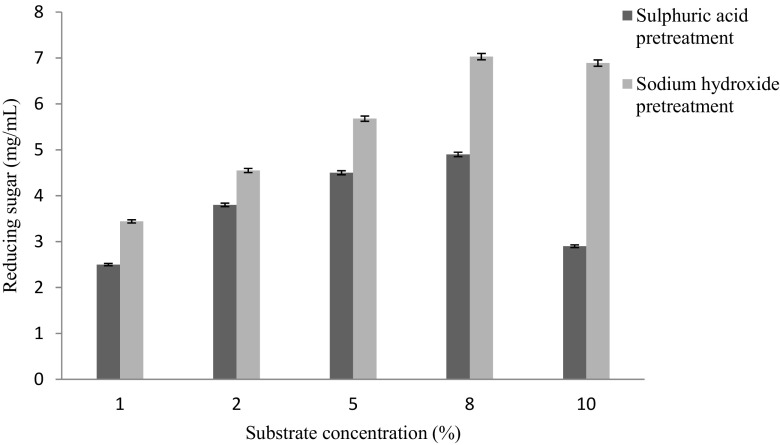
Effect of substrate concentration (%) on thermochemical pretreatment of lignocellulosic biomass (constant values: acid/alkali concentration: 0.2 M, temperature, 120 °C, incubation time: 20 min)

**Fig. 5:**
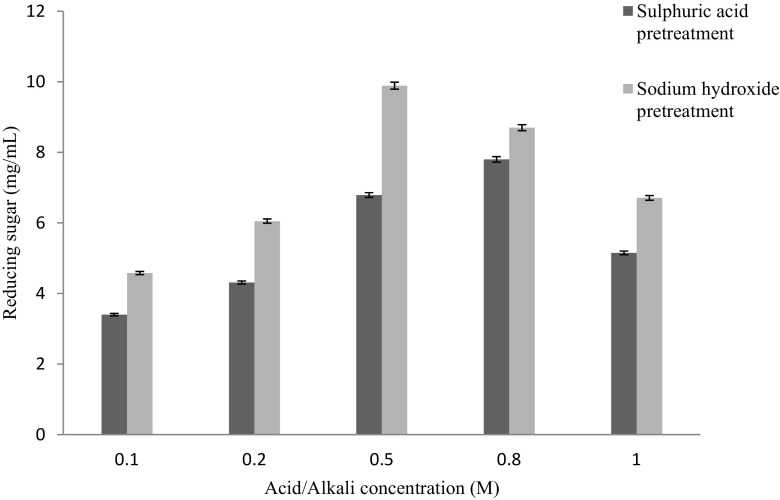
Effect of acid/alkali concentration (M) on thermochemical pretreatment of lignocellulosic biomass (constant values: substrate concentration: 5 %, temperature, 120 °C, incubation time: 20 min)

**Fig. 6:**
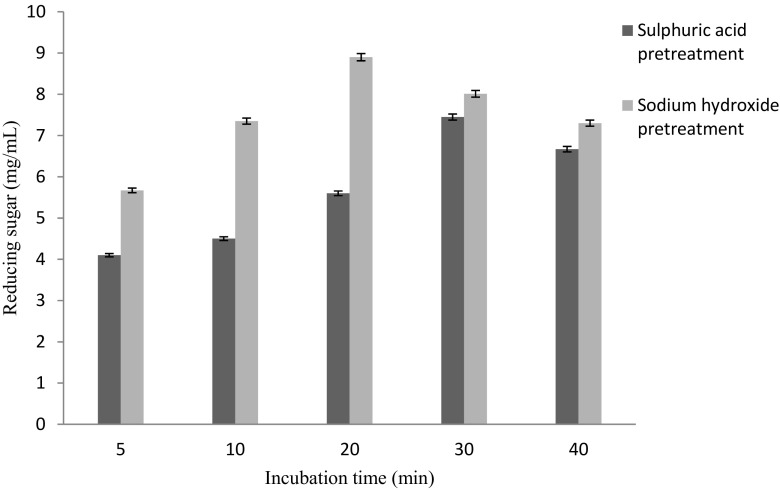
Effect of incubation time (min) on thermochemical pretreatment of lignocellulosic biomass (constant values: substrate concentration 5 %, acid/alkali concentration: 0.5 M, and temperature, 120 °C)

In the present study dilute acid and alkaline pretreatment of lignocellulosic biomass was carried out under varying conditions of substrate concentration (1, 2, 5, 8 and 10 %), dilute acid/alkaline concentration (0.1, 0.2, 0.5, 0.8 and 1 M) and incubation time (5, 10, 20, 30 and 40 min). Figure 4 showed the effect of different substrate concentration on thermochemical pretreatment of lignocellulosic biomass. It showed maximum reducing sugar (7.03 mg/mL) was obtained in case of biomass pretreatment with 0.2 M sodium hydroxide at 8 % substrate concentration, 20 min incubation time and 120 °C temperature. Further increase or decrease in substrate concentration during thermochemical pretreatment, reducing sugar yield was decreased. Similar type of observation was reported by Hong et al. (2012) and McIntosh and Vancov (2011) in case of biomass pretreated with dilute phosphoric acid and sodium hydroxide, respectively. Figure 5 demonstrated the effect of acid/alkaline concentration on thermochemical pretreatment of biomass. It showed maximum reducing sugar (9.89 mg/mL) was obtained in case of biomass pretreated with 0.5 M sodium hydroxide using 8 % substrate concentration, 20 min incubation time and 120 °C temperature. Wang et al. (2010) reported sodium hydroxide pretreatment of Bermuda grass. Authors reported maximum reducing sugar production when the biomass was pretreated with 0.19 M sodium hydroxide for 15 min at 121 °C. The variation in optimum sodium hydroxide concentration was might be due to different biomass was used for pretreatment. Effect of incubation time on thermochemical pretreatment of biomass has been demonstrated in Fig. 6. The maximum reducing sugar (8.9 mg/mL) was obtained when biomass was treated with 0.5 M sodium hydroxide at 8 % substrate concentration, 20 min incubation time and 120 °C temperature. Choi et al. (2013) reported optimum sodium hydroxide pretreatment of empty fruit bunch at 3 % sodium hydroxide concentration, 11 min 20 s incubation time and 140 °C temperature. The above results showed that optimum thermochemical pretreatment condition was 0.5 M sodium hydroxide concentration, 8 % substrate concentration, 20 min incubation time and 120 °C temperature. The maximum reducing sugar yield from pretreated biomass (under optimum conditions) was 398.0 mg/g dry biomass within 24 of enzymatic hydrolysis. Kshirsagar et al. (2015) reported maximum reducing sugar yield (359 mg/g dry biomass) from dilute acid pretreated rice straw after 72 h enzymatic hydrolysis. Ioelovich and Morag (2012) reported 82 % reducing sugar yield from mild acid and alkaline pretreated biomass after 48 h of enzymatic hydrolysis. The present study showed higher reducing sugar production from mixture of biomass (wheat straw and cotton stalk) within short incubation time (24 h). This study has also established that the cheap substrates could effectively be used for ethanol production through further process optimization of simultaneous saccharification and fermentation.

### FTIR analysis of lignocellulosic biomass

**Fig. 7:**
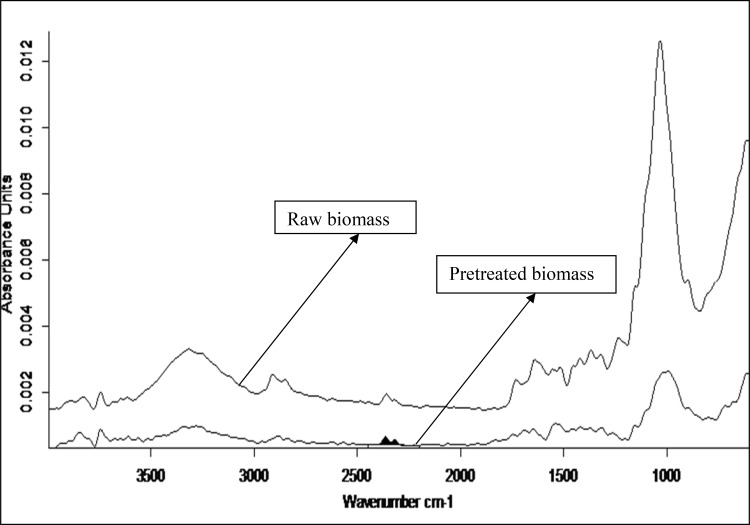
FTIR spectra of lignocellulosic biomass (raw and pretreated)

FTIR spectral characterization of raw and sodium hydroxide pretreated lignocellulosic biomass was carried out in the region range of 600–4000 cm^−1^. Figure 7 showed the IR spectra of raw and pretreated biomass. The peak around 1032 cm^−1^ corresponds to aromatic C–H deformation (present in lignin), C–O deformation in primary alcohol and stretching of non conjugated C=O bond (lignin and hemicelluloses). The band around 1693 cm^−1^ corresponds to un-conjugated C–O stretching (present in lignin) and band around 2838–2911 cm^−1^ corresponds to C–H stretching of lignin polymer. The band around 3318 and 3815 cm^−1^ corresponds to O–H stretching of lignin and hemicelluloses polymer. Table 4 summarizes the absorbance band and corresponding functional groups present in lignocellulosic biomass. All the band intensities were reduced after sodium hydroxide pretreatment, indicates the degradation of lignin polymer during pretreatment. The reduced intensity indicates cleavage of lignin side chains. The above result highlight the effectiveness of dilute alkaline pretreatment of lignocellulosic biomass for enzymatic hydrolysis.

**Table 4:** FTIR band and corresponding groups present in lignocellulosic biomass (Bodirlau et al. 2009; Xu et al. 2013; Nakashima et al. 2016)

Wave number (cm^−1^)	Functional group	Remark
1032	C–H deformation	Present in lignin
1693	Aromatic ring	Present in lignin
1745	Stretching asymmetric and symmetric vibration of CO_2_	Present in lignin and cellulose
2360	C–H stretching	Present in lignin
2884		
3318, 3815	O–H stretching	Present in lignin and hemicellulose

### SEM analysis of lignocellulosic biomass

**Fig. 8:**
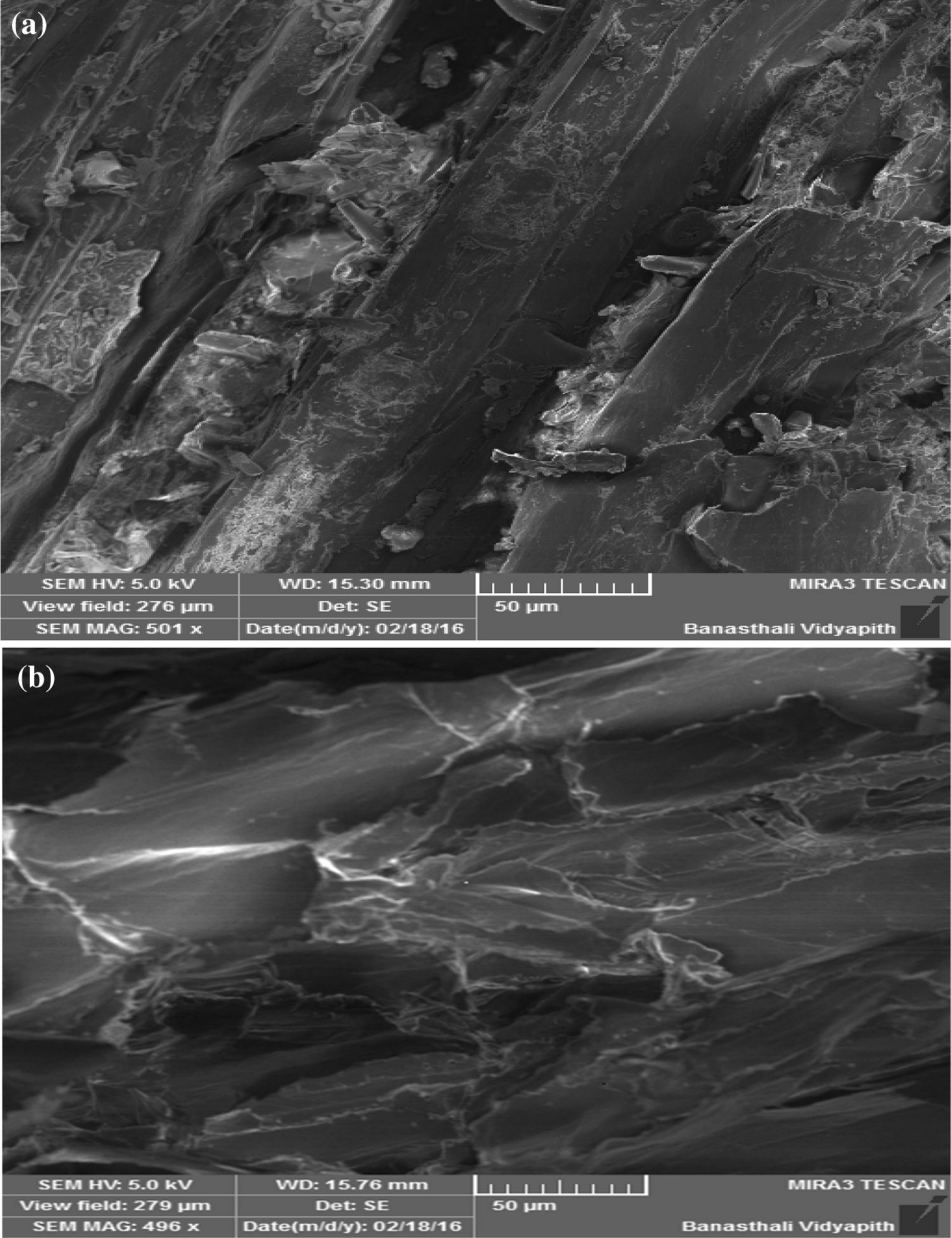
SEM image of lignocellulosic biomass (raw and pretreated)

Figure 8 showed the SEM picture of raw and pretreated (sodium hydroxide pretreated lignocellulosic biomass. It showed after pretreatment the cell wall structure of lignocellulosic biomass was degraded. This finding demonstrated that degradation of lignin and hemicelluloses during pretreatment cause distortion of cell wall structure of lignocellulosic biomass. Similar types of observation were reported earlier (Cui et al. 2012; Wei et al. 2015). The SEM analysis demonstrated the effectiveness of dilute alkaline pretreatment for efficient saccharification of lignocellulosic biomass.

### TLC analysis of sugars present in saccharified sample of pretreated biomass

Sugars present in the saccharified sample were analyzed by calculating their *R*_f_ value (Table 5). The result suggested the presence of different sugars (glucose, xylose, mannose, maltose) in the saccharified sample.

**Table 5:** R_f_ values of sugars present in saccharified sample

Sugars	*R*_f_ values of standard sugars	*R*_f_ values of sugars present in saccharified sample
Xylose	0.872	0.872
Ribose	0.672	–
Arabinose	0.590	–
Mannose	0.551	0.551
Glucose	0.456	0.456
Maltose	0.321	0.321

## Conclusion

The present study deals with optimization of cellulase production under submerged fermentation using natural medium and waste paper. Maximum cellulase production (0.53 IU/mL) was obtained within 3 days of incubation time. The produced cellulase was applied for hydrolysis of dilute acid and alkaline pretreated biomass. It showed maximum reducing sugar yield of 398.0 mg/g dry biomass was obtained from dilute alkaline pretreated biomass (under sodium hydroxide concentration of 0.5 M, substrate concentration of 8 %, temperature of 120 °C and incubation time of 20 min). Further effectiveness of dilute alkaline pretreatment was analyzed through FTIR and SEM study.
